# Degradable Self-Destructive Redox-Responsive System Based on Mesoporous Organosilica Nano-Vehicles for Smart Delivery of Fungicide

**DOI:** 10.3390/nano12234249

**Published:** 2022-11-29

**Authors:** You Liang, Sijin Wang, Yijia Yao, Siwen Yu, Ao Li, Yuanfeng Wang, Jiehui Song, Zhongyang Huo

**Affiliations:** Jiangsu Co-Innovation Center for Modern Production Technology of Grain Crop, Jiangsu Key Laboratory of Crop Genetics and Physiology, Yangzhou University, NO.48 Wenhui East Road, Yangzhou 225009, China

**Keywords:** mesoporous organosilica, gallic-acid–Fe(III) complexes, prochloraz, redox-responsive, self-destructive, *Magnaporthe oryzae*

## Abstract

The development of stimuli-responsive controlled release formulations is a potential method of improving pesticide utilization efficiency and alleviating current pesticide-related environmental pollution. In this study, a self-destruction redox-responsive pesticide delivery system using biodegradable disulfide-bond-bridged mesoporous organosilica (DMON) nanoparticles as the porous carriers and coordination complexes of gallic acid (GA) and Fe(III) ions as the capping agents were established for controlling prochloraz (PRO) release. The GA–Fe(III) complexes deposited onto the surface of DMON nanoparticles could effectively improve the light stability of prochloraz. Due to the decomposition of GA–Fe(III) complexes, the nano-vehicles had excellent redox-responsive performance under the reducing environments generated by the fungus. The spreadability of PRO@DMON–GA–Fe(III) nanoparticles on the rice leaves was increased due to the hydrogen bonds between GA and rice leaves. Compared with prochloraz emulsifiable concentrate, PRO@DMON–GA–Fe(III) nanoparticles showed better fungicidal activity against *Magnaporthe oryzae* with a longer duration under the same concentration of prochloraz. More importantly, DMON–GA–Fe(III) nanocarriers did not observe obvious toxicity to the growth of rice seedlings. Considering non-toxic organic solvents and excellent antifungal activity, redox-responsive pesticide controlled release systems with self-destruction properties have great application prospects in the field of plant disease management.

## 1. Introduction

In modern agriculture, pesticides play an irreplaceable role in crop yield and pest management [1,2]. However, about 0.1% of the traditionally used pesticides reach the desired target site of organisms, while 99.9% are lost into the surrounding environment [3]. Pesticide application in the field is inefficient due to runoff, biodegradation, ultraviolet (UV) degradation, leaching, and rapid evaporation, which not only increases the cost of pesticide application, but also has adverse effects on the environment [4,5]. At present, controlled release pesticide delivery systems have been developed to improve the utilization efficiency of active ingredients by increasing bioavailability, reducing photolysis, and prolonging durability [6,7,8]. Conventional controlled release pesticide systems based on microencapsulation and clays can only release active ingredients slowly through molecular diffusion rather than achieve on-demand release at temporal and spatial resolutions [9,10,11,12]. Therefore, developing a precisely controlled release system is urgent to guarantee spatiotemporal pesticide release [13].

In recent years, many efforts have been devoted to developing stimuli-responsive nanomaterials with the hope that they can be designed as effective pesticide delivery vehicles [14,15,16]. A variety of endogenous (including pH values [17], enzyme [18], and redox [19]) or exogenous stimuli (including temperature [20], light [21], magnetic field [22], and ultrasound [23]) have been used to induce chemical reactions in stimuli-responsive nanocarriers for realizing on-demand pesticide delivery. Among these various stimuli-responsive nanomaterials, disulfide-bond-bridged mesoporous organosilica (DMON) nanoparticles with redox-responsive properties are attracting much attention due to their excellent biocompatibility and degradable properties in reducing environments [18,24,25]. The disulfide bond in the nanocarriers framework is one of the most biologically effective cleavable linkers in biology, which can be degraded and cleaved in the presence of glutathione [26]. The conventional mesoporous silica nanoparticles show a lower pesticide release rate and cannot degrade in the reduction environment in comparison with redox-responsive biodegradable DMON nanoparticles. Therefore, the DMON nanoparticles are optimal candidates as supporting materials to control pesticide release.

Gallic acid (GA) is a naturally occurring secondary metabolite that is widely present in plant tissues and plays an important role in plant defense against pathogens and insects [27,28,29,30]. As a water-soluble natural polyphenolic compound, the catechol groups of GA can react with Fe(III) ions to produce stable, surface-confined amorphous films on solid substrates [31,32,33]. The metal–phenolic network formed by the self-assembly of the strong metal–phenol complex interface can effectively block porous pores of nanocarriers and inhibit the migration of pesticide molecules [34,35,36]. In addition, the cross-linking process between GA and Fe(III) ions is rapid based on the wet chemistry method, the reaction process is fast and free of contaminants or toxic substances, which is a green and visible encapsulation strategy [37]. Therefore, GA–Fe(III) complexes can be employed as a supramolecular capping agent to block the pores of DMON nanoparticles.

Rice (*Oryza sativa* L.) is a crucial cultivated crop in the world, which supports over half of the global population [38]. As the most destructive disease on rice, rice blast caused by ascomycetous fungus (*Magnaporthe oryzae*) can induce an annual rice production loss of 10–30% (up to 50% in disease epidemics) [39,40]. It has been reported that *Magnaporthe oryzae* has an efficient antioxidant defense system, which can produce glutathione during host plant infection to neutralize reactive oxygen species produced by host defense responses [41,42]. Microenvironmental variations at disease-occurring sites facilitate the development of multiple-stimuli-responsive nanocarriers for on-demand and on-target pesticide release [43]. The loaded pesticide molecules can be precisely released to the target site due to the influence of the microenvironment generated by the pathogen infection site [44]. Thus, combining the pathogen infection process with the targeted pesticide release technique is an efficient approach to manage plant diseases. 

As an inhibitor of the enzyme lanosterol 14α-demethylase, prochloraz (N-propyl-N-[2-(2,4,6-trichlorophenoxy)ethyl]imidazole-1-carboxamide) has excellent antifungal activity against *Sclerotinia sclerotiorum* [45], *Penicillium digitatum* [46], *Lasiodiplodia theobromae* [47], *Rhizoctonia solani* [48], *Fusarium fujikuroi* [49], and *Magnaporthe oryzae* [50]. The most commonly used prochloraz formulation types include emulsifiable concentrate and oil-in-water emulsion because of its low solubility in water (about 26.5 μg/mL) [51,52]. However, prochloraz is unstable under UV and natural light, which leads to its short-term efficiency and low utilization rate [53]. To address these issues, prochloraz can be incorporated into the stable and biocompatible nanoparticles, thus enhancing photostability, increasing water solubility, and prolonging the effective duration of active ingredients. 

Herein, the self-destructive redox-responsive biodegradable nano-vehicles were established using DMON nanoparticles as porous carriers and metal–phenolic networks as gatekeepers to deliver prochloraz for rice disease management. The redox environment caused by *Magnaporthe oryzae* infection could trigger degradation of the DMON nanoparticles and metal–phenolic networks, and the prochloraz released from PRO@DMON–GA–Fe(III) nanoparticles would induce the death of the pathogen. The characterization, release profiles, release mechanism, UV-shielding properties, spreadability, fungicidal activities, and biosafety of the prepared PRO@DMON–GA–Fe(III) nanoparticles were comprehensively investigated. 

## 2. Experimental

### 2.1. Materials

Prochloraz technical (97% purity) was purchased from Hubei Jiufenglong Chemical Co., Ltd. (Wuhan, China). The 25% prochloraz emulsifiable concentrate was supplied by Sichuan Runer Technology Co., Ltd. (Chengdu, China). Triethylamine (TEA), tetraethyl orthosilicate (TEOS), and glutathione were received from Sinopharm Chemical Reagent Beijing Co., Ltd. (Beijing, China). Bis(triethoxysilyl propyl) disulfide (BTESPT), cetyltrimethylammonium bromide (CTAB), gallic acid (GA), sodium hydroxide (NaOH), and ferric chloride hexahydrate (FeCl_3_·6H_2_O) were supplied by Shanghai Aladdin Biochemical Technology Co., Ltd. (Shanghai, China). Methanol, ethanol, acetic acid, and concentrated hydrochloric acid (HCl, 37%) were purchased from Sinopharm Chemical Reagent Beijing Co., Ltd. (Beijing, China). Methanol (HPLC grade) was ordered from Adamas Beta Co., Ltd. (Shanghai, China). Deionized water (18 MΩ/cm) was prepared using a Milli-Q water purification system (Millipore, Milford, MA, USA). 

### 2.2. Preparation of PRO@DMON–GA–Fe(III) Nanoparticles

#### 2.2.1. Synthesis of Disulfide-Bridged Mesoporous Organosilica (DMON) Nanoparticles

In a typical synthesis, 0.25 mL of TEA and 0.48 g of CTAB were dissolved in 15.5 mL of ethanol. This solution was then introduced into 94 mL of deionized water and mixed for 30 min at 80 °C. Then, another mixture (2.0 mL of TEOS and 0.8 mL of BTESPT) was added to the surfactant solution dropwise. The obtained mixture was heated at 80 °C for another 6 h. Next, under centrifugation at 20,000 rpm for 15 min, the resulting white products were obtained and fully washed using ethanol and deionized water. To remove the surfactant in the pores of DMON nanoparticles, the obtained products were redispersed in the acidic ethanol (3 mL of HCl and 100 mL of ethanol) and then refluxed at 80 °C for 12 h. The reflux was repeated at least four times. The CTAB-free DMON nanoparticles were collected by centrifugation, washed with ethanol, and dried under vacuum at 60 °C for 12 h.

#### 2.2.2. Synthesis of PRO@DMON–GA–Fe(III) Nanoparticles

Approximately 400 mg of prochloraz was added to 10 mL of methanol, and then 200 mg of DMON nanoparticles were suspended in the prepared pesticide solution. The obtained prochloraz-loaded hybrid nanoparticles (PRO@DMON nanoparticles) were washed with deionized water several times after stirring in the dark at room temperature for 48 h. The precipitate was vacuum-dried at 60 °C for 12 h. After that, 20 mg of PRO@DMON nanoparticles were dispersed in 2 mL of deionized water. Approximately 400 μL of FeCl_3_·6H_2_O solution (30 mM) and 800 μL of GA solution (15 mM) were also added. After vortexing for 30 s, the suspension pH was adjusted using 40 μL of NaOH solution (0.5 M), which was followed by vortexing for 60 s. The PRO@DMON–GA–Fe(III) nanoparticles were collected by centrifugation, washed with deionized water, and dried under vacuum at 60 °C for 12 h.

### 2.3. Characterization

The morphology of the porous structure of the prepared nanoparticles was characterized using a Philips Tecnai 12 transmission electron microscope (TEM) at an acceleration voltage of 120 kV (Phillips, Eindhoven, The Netherlands). The elemental mapping of the as-synthesized samples was conducted using high-resolution TEM (HRTEM, Tecnai G2 F30, FEI, Hillsboro, Oregon USA) equipped with an energy-dispersive X-ray spectroscopy (EDX) system. The elemental composition and chemical state of the samples were characterized by X-ray photoelectron spectroscopy (XPS, Thermo ESCALAB 250Xi, Thermo-Fisher, Waltham, MA, USA). A Tensor 27 Fourier transform spectrometer (FTIR) (Thermo-Fisher, Waltham, MA, USA) was used to analyze various functionalized nanoparticles. A TriStar II 3020 analyzer was used to conduct the nitrogen adsorption/desorption experiments (Micromeritics Instrument Corporation, Norcross, USA). The Brunauer–Emmett–Teller (BET) theory and Barret–Joyner–Halenda (BJH) were adopted to calculate the surface area and pore size of the samples, respectively. The contact angle and adhesion work on rice blades were measured using an SL-200B optical contact-angle goniometer (Kino Industrial Co., Ltd., New York, NY, USA).

### 2.4. Biodegradation Assays of DMON Nanoparticles

Typically, approximately 2 mg of DMON nanoparticles was dispersed in deionized water with 10 mM of glutathione. The mixture was stirred at room temperature for 10 d. At pre-determined time points (day 0, 2, 5, and 10), the samples were obtained to investigate the structural changes using TEM.

### 2.5. Pesticide Loading Content and Controlled Release Kinetics

#### 2.5.1. Pesticide Loading Content

In order to evaluate the prochloraz content in the PRO@DMON–GA–Fe(III) nanoparticles, the nanoparticles were added to a methanol–water mixture solution (50:50, *v*/*v*), which contains 100 mM of glutathione. The concentration of prochloraz released from PRO@DMON–GA–Fe(III) nanoparticles was evaluated using a Waters high-performance liquid chromatography (HPLC) (Milford, MA, USA). The HPLC was equipped with a diode array detector with an InertSustain C_18_ analytical column (5 μm, 250 × 4.6 mm, GL Sciences Inc., Tokyo, Japan) maintained at 35 °C. The aqueous mobile phase contained 70% methanol and 30% ultra-pure water with 0.1% acetic acid. The injection volume of the samples was 20 μL and the mobile phase flow rate was 1 mL/min. The solvents were then filtered using a 0.45 μm membrane filter for HPLC measurements. The prochloraz loading efficiency of the nanoparticles was calculated according to Equation (1).

(1) $$\mathrm{Loading}\;\mathrm{capacity}\;\left(\%\right)=\frac{\mathrm{amount}\;\mathrm{of}\;\mathrm{prochloraz}\;\mathrm{encapsulated}\;\mathrm{in}\;\mathrm{the}\;\mathrm{nanoparticles}}{\mathrm{total}\;\mathrm{amount}\;\mathrm{of}\;\mathrm{PRO}@\mathrm{DMON}–\mathrm{GA}–\mathrm{Fe}\left(\mathrm{III}\right)\;\mathrm{nanoparticles}}\times 100$$

#### 2.5.2. Controlled Release Kinetics

The influence of glutathione on the prochloraz release behavior of PRO@DMON–GA–Fe(III) nanoparticles was analyzed using the HPLC method. Approximately 1.5 mL of PRO@DMON–GA–Fe(III) nanoparticles dispersion solution at a concentration of 10 mg/mL was transferred into the dialysis bag. Then, the dialysis bag was immersed in 48.5 mL of methanol–water mixture (30:70, *v*/*v*) with different concentrations of glutathione (0, 2, and 10 mM) under slow magnetic stirring in the dark condition. At each specific time, 1 mL of the solution was extracted and an equal volume of methanol–water mixture (30:70, *v*/*v*) containing glutathione was replenished. Each treatment was repeated three times.

The release behaviors of prochloraz from the PRO@DMON–GA–Fe(III) nanoparticles suspension were evaluated using the zero-order (Equation (2)), first-order (Equation (3)), Ritger–Peppas (Equation (4)) and Higuchi (Equation (5)) models, respectively.

(2) $$\mathrm{Zero}\text{-}\mathrm{order}\;\mathrm{model}:\;\frac{M_{t}}{M_{z}}=kt$$

(3) $$\mathrm{First}\text{-}\mathrm{order}\;\mathrm{model}:\;\;\frac{M_{t}}{M_{z}}=1-e^{-kt}$$

(4) $$\mathrm{Ritger}–\mathrm{Peppas}\;\mathrm{model}:\;\frac{M_{t}}{M_{z}}=kt^{n}$$

(5) $$\mathrm{Higuchi}\;\mathrm{model}:\;\frac{M_{t}}{M_{z}}=kt^{\frac{1}{2}}$$

where *k* is the kinetic constant and relies on the combined characteristics of prochloraz and DMON–GA–Fe(III) nanoparticles system; *M_t_*/*M_z_* represents the percentage of prochloraz released from PRO@DMON–GA–Fe(III) nanoparticles at time *t*; *n* is a diffusional exponent to characterize the release mechanism of prochloraz from the nanoparticles.

### 2.6. Photolysis Test

To investigate the differences in photo-stability between the samples, PRO@DMON–GA–Fe(III) nanoparticles, prochloraz emulsifiable concentrate and prochloraz technical at the same prochloraz concentration were dispersed in a water–methanol mixture (70:30, *v*/*v*), respectively. The resultant solutions were transferred into quartz tubes at a distance of 20 cm from a 32 W germicidal UV lamp (254 nm). At 0, 0.5, 1, 2, 3, 5, 7, 10, 13, 16, 20, 24, 36, and 48 h, the prochloraz content in each quartz tube was analyzed using HPLC. The collected samples were filtered through a 0.45 μm membrane filter. Each treatment was repeated three times. The cumulative retention rate of prochloraz in the samples was calculated using Equation (6).

(6) $$\mathrm{Cumulative}\;\mathrm{retention}\;\mathrm{rate}\;\left(\%\right)=\frac{C_{t}}{C_{0}}\times 100$$

The prochloraz photodegradation of the samples was described using the pseudo-first-order kinetics Equation (7).

(7) $$\ln\frac{C_{t}}{C_{0}}=-kt$$

where *C*_0_ is the initial amount prochloraz in PRO@DMON–GA–Fe(III) nanoparticles; *C_t_* is the retention amount of prochloraz in the system at time t; and *k* is the rate constant.

### 2.7. Spreadability of PRO@DMON–GA–Fe(III) Nanoparticles on Rice Blades

The adhesion work and contact angle were investigated in order to reveal the spreadability of PRO@DMON–GA–Fe(III) nanoparticles on rice blades. The rice leaf was sliced and securely attached to glass slides. Then, a microinject was used to deposit 5 μL of PRO@DMON–GA–Fe(III) nanoparticle aqueous suspension on the rice blade. The contact angle images and values were recorded by the sessile-drop method after 5 s. PRO@DMON nanoparticles were used as controls. 

### 2.8. Fungicidal Activity

The antifungal activities of PRO@DMON–GA–Fe(III) nanoparticles and prochloraz emulsifiable concentrate against *Magnaporthe oryzae* were assayed by mycelium growth rate method. Different concentrations of PRO@DMON–GA–Fe(III) nanoparticles and prochloraz emulsifiable concentrate were added into the molten potato dextrose agar (PDA) medium, where all the concentrations were determined by the mass of prochloraz technical. Then, 20 mL of the medium containing prochloraz (0.125, 0.25, 0.5, 1.0, 2.0, and 4.0 mg/L) was placed into the sterilized Petri dish with a diameter of 9 cm. A 6 mm diameter mycelium disc which was 10 days old was cut from the edge of the culture media and placed in the center of the above medicated medium after solidification for 2 h. The medium containing sterile water or blank DMON–GA–Fe(III) nanoparticles was also inoculated and used as controls. The PDA agar plates were incubated at 25 °C in the dark condition for 14 days. Finally, the mean diameter of the radial mycelial growth was measured three times in different perpendicular directions. All treatments were performed in triplicate. The percentage of mycelium growth inhibition rate was calculated according to the Equation (8).

(8) $$\mathrm{Mycelium}\;\mathrm{growth}\;\mathrm{inhibition}\;\mathrm{rate}\;\left(\%\right)=\left(1-\frac{D_{t}}{D_{c}}\right)\times 100$$

where *D_t_* and *D_c_* are the colony diameter of the treatment group and the control group at day 14, respectively.

### 2.9. Safety Assay

Rice seeds (*Oryza sativa* L. cv Xiangliangyou 900) were surface sterilized with 75% ethanol (*v*/*v*) for 5 min, then treated with 5% sodium hypochlorite (5% available chlorine) for 5 min and washed with deionized water several times. After soaking for 48 h in ultra-pure water, fifteen seeds were placed onto the sterilized Petri dishes (9 cm in diameter) containing double filter papers. Approximately 5 mL of blank DMON–GA–Fe(III) nanoparticles with different concentrations (0, 50, 100, 200, 400, and 800 mg/L) were added into each Petri dish. The treated rice seeds were cultivated in a growth incubator in the dark. The germination rate, root length, shoot length, root number, and fresh weight of the rice seedling were recorded after 7 days. Each treatment was repeated five times.

### 2.10. Data Analysis

SPSS 23.0 was used to conduct the statistical analyzes (SPSS, Chicago, IL, USA). The TEM images were analyzed using ImageJ software to determine nanoparticle diameter. The probit regression model was used to evaluate *EC*_50_ values. The Duncan’s multiple range test (*p* < 0.05) was used for data analysis, and the experimental data were expressed as the mean ± standard error (SE). The graphs were obtained using Origin 2022b (learning edition). Each experiment was conducted at least three times.

## 3. Results and Discussion

### 3.1. Preparation and Characterization of PRO@DMON–GA–Fe(III) Nanoparticles

**Figure 1:**
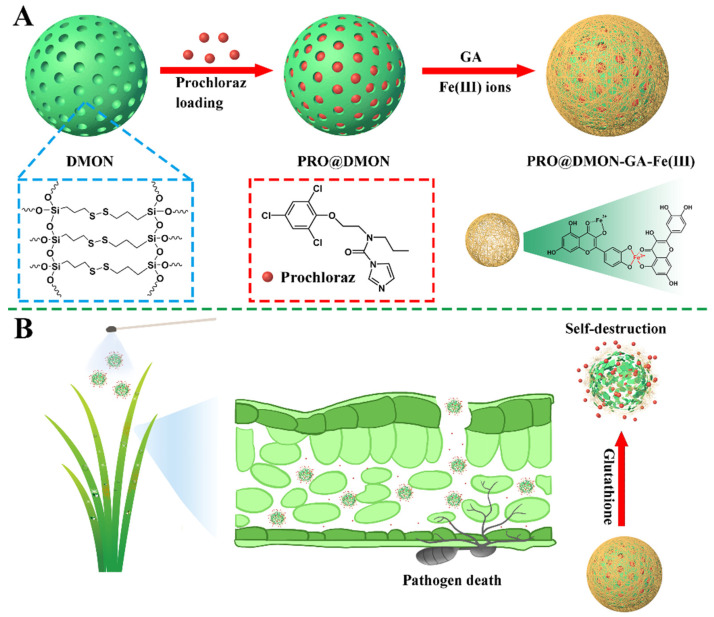
The mechanism for preparation of PRO@DMON–GA–Fe(III) nanoparticle (**A**). The triggered release mechanism of prochloraz from PRO@DMON–GA–Fe(III) nanoparticles (**B**).

Figure 1A shows the detailed preparation procedures of PRO@DMON–GA–Fe(III) nanoparticles. Firstly, the DMON nanoparticle was synthesized using a modified sol–gel method (with TEA as the catalyst, TEOS and BTESPT as the silicon source, and CTAB as the structure-directing agents). After extracting CTAB from the pores of the DMON nanoparticle, prochloraz molecules were loaded into the DMON nanoparticle to prepare a pesticide-loaded hybrid nanoparticle (PRO@DMON nanoparticle). Finally, the (GA–Fe(III)) complexes were deposited on the surface of the hybrid nanoparticle through the coordination between GA and Fe(III) ions, and the PRO@DMON–GA–Fe(III) nanoparticle was obtained. 

**Figure 2:**
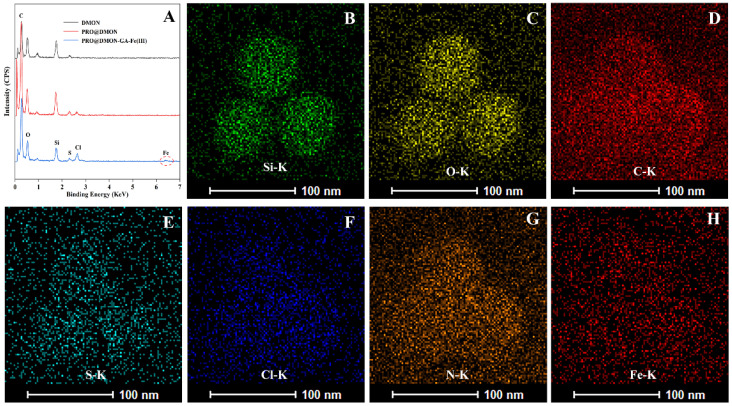
EDX spectra of DMON nanoparticles, PRO@DMON nanoparticles and PRO@DMON–GA–Fe(III) nanoparticles (**A**). TEM mapping of PRO@DMON–GA–Fe(III) nanoparticles (**B**–**H**).

The EDX spectroscopy and TEM mapping were used to analyze the elemental composition differences among DMON nanoparticles, PRO@DMON nanoparticles, and DMON–GA–Fe(III) nanoparticles. Compared with blank DMON nanoparticles, a new peak attributed to chlorine can be observed in PRO@DMON nanoparticles, proving the successful loading of prochloraz. After further deposition of GA–Fe(III) complexes on the surface of PRO@DMON nanoparticles, the Fe peak was observed at the binding energy around 6.4 keV (Figure 2A). In addition, the element mappings (Figure 2B–F) of DMON–GA–Fe(III) nanoparticles demonstrate that the O, Si, S, Fe, and C elements were uniformly distributed in the nanoparticles. The above results indicate the successful loading of pesticide and the deposition of GA–Fe(III) complexes in DMON nanoparticles.

**Figure 3:**
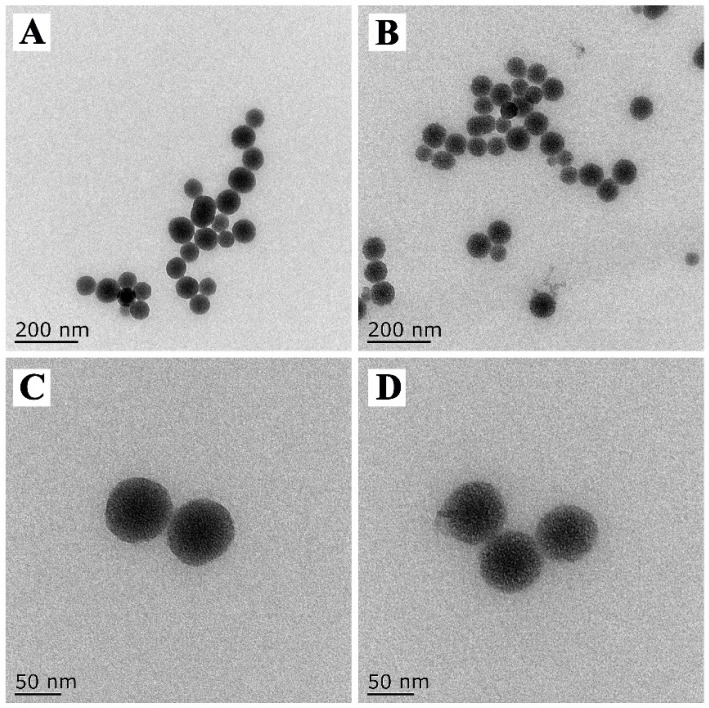
TEM images of DMON nanoparticles (**A**,**C**) and PRO@DMON–GA–Fe(III) nanoparticles (**B**,**D**).

The prepared DMON nanoparticles had a homogeneous spherical morphology with good symmetry, and a uniform diameter of approximately 80 nm (Figure 3A). The enlarged TEM image indicated that the pores of the sphere were well-defined central-radial structures (Figure 3C). After prochloraz loading and GA–Fe(III) complexes deposition, the mesoporous DMON nanoparticles arrays were partly hidden indicating the successful preparation of PRO@DMON–GA–Fe(III) nanoparticles (Figure 3B,D). 

**Figure 4:**
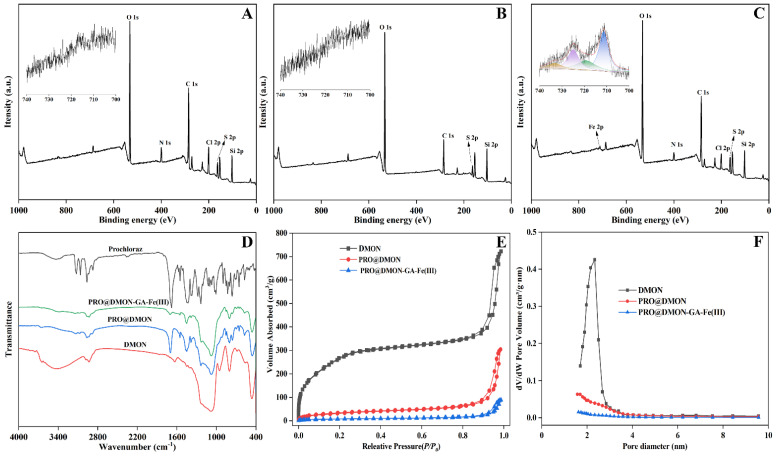
XPS spectra of DMON nanoparticles (**A**), PRO@DMON nanoparticles (**B**), and PRO@DMON–GA–Fe(III) nanoparticles (**C**). FTIR spectra of prochloraz, DMON nanoparticles, PRO@DMON nanoparticles, and PRO@DMON–GA–Fe(III) nanoparticles (**D**). Nitrogen adsorption–desorption isotherms (**E**), and BJH pore size (**F**) of DMON nanoparticles, PRO@DMON nanoparticles, and PRO@DMON–GA–Fe(III) nanoparticles.

XPS spectra showed that the peaks of DMON nanoparticles at binding energies of 532.79, 399.94, 285.02, and 103.25 eV corresponded to Si 2p, C 1s, N 1s, and O 1s, respectively. This result indicates the successful synthesis of disulfide-bridged mesoporous organosilica nanoparticles (Figure 4A). After prochloraz loading, two new characteristic peaks attributed to N and Cl elements appeared at 399.90 and 200.47 eV, respectively, indicating the existence of prochloraz in the DMON nanocarriers (Figure 4B). For PRO@DMON–GA–Fe(III) nanoparticles, the major peak at 711.19 eV in the Fe 2p spectra confirmed the presence of Fe(III) ions (Figure 4C). The high-resolution Fe 2d XPS spectrum of PRO@DMON–GA–Fe(III) nanoparticles in Figure 4C indicated that the two main peaks at 711.35 and 724.35 eV belonged to the binding energies of 2p3/2 and the 2p1/2 region of Fe(III) ions, respectively, suggesting the successful deposition of the GA–Fe(III) complexes on the surface of the PRO@DMON nanoparticles [54].

To further verify the formation processes of PRO@DMON–GA–Fe(III) nanoparticles, FTIR spectra of the samples were analyzed. The DMON nanoparticles exhibited strong absorption peaks at 1084, 803, and 464 cm^−1^, respectively (Figure 4D). These peaks can be attributed to asymmetric stretching and the stretching of Si−O−Si bonds. The peaks at 3434 and 1633 cm^−1^ were due to adsorbed water molecules or –OH groups on the surface of DMON nanoparticles. After loading prochloraz to the nanocarriers, two new peaks were displayed at 1698 and 1451 cm^−1^, which were attributed to the stretching vibrations of C=O and C=N [55,56]. Next, the resultant hybrid nanoparticles were deposited with GA–Fe(III) complexes. The peak intensities of the prochloraz in PRO@DMON–GA–Fe(III) nanoparticles at 1698 and 1451 cm^−1^ were decreased, indicating the GA–Fe(III) complexes wrapped on the surface of the PRO@DMON nanoparticles successfully. 

Nitrogen adsorption–desorption isotherms during pesticide loading and modification were characterized to investigate the variations of pore size distribution and BET-specific surface area. The DMON nanoparticles showed a typical type IV isotherm based on the hysteresis loop (Figure 4E), indicating their well-defined mesoporous structure. The specific surface area, average pore size, and pore volume were 1020.81 m^2^/g, 2.33 nm, and 1.13 cm^3^/g, respectively. After prochloraz molecules loading and GA–Fe(III) complexes deposition, the nanoparticles showed decreasing pore volume and pore size (Figure 4F and Table 1). These results validate that the pesticide loading was successful, and the pores were completely blocked by GA–Fe(III) complexes.

### 3.2. Reducibility-Responsive Biodegradability Assay

**Figure 5:**
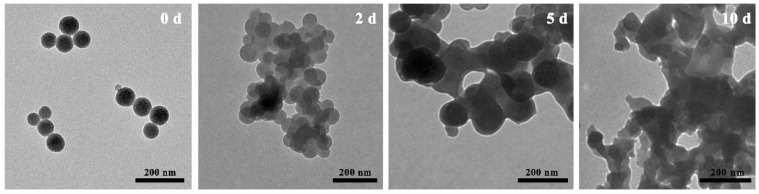
Degradation behaviors of DMON nanoparticles in 10 mM glutathione solution at 0, 2, 5, and 10 days.

To investigate the reducibility-responsive biodegradability of the as-synthesized DMON nanoparticles, the morphological changes of the samples over time under the reducing environment were also monitored (Figure 5). The glutathione was adopted to simulate the reduction condition at the localized necrosis site of plants. On the second day of the experiment, the spherical morphologies were mainly retained. With the incubation time extended to five days, a slight number of DMON nanoparticles had spherical morphologies. On day 10, all DMON nanoparticles were severely damaged. These results indicated that the gradual degradation of DMON could be achieved by extending the incubation time in reduction environments [57,58]. The reducibility-responsive property of the nanoparticles facilitates their use as pesticide carriers for the on-demand release of active ingredients.

### 3.3. Pesticide Loading and Controlled Release Kinetics

The prochloraz loading efficiency of PRO@DMON–GA–Fe(III) nanoparticles was determined as 13.8% using HPLC. The mechanism of prochloraz-triggered release from PRO@DMON–GA–Fe(III) nanoparticles is shown in Figure 1B. When rice plants were infected by pathogens, the reductive environment secreted by *Magnaporthe oryzae* could induce the decomposition of the capped materials (GA–Fe(III) complexes) and the porous carriers (DMON nanoparticles), which would trigger the release of prochloraz and lead to the death of *Magnaporthe oryzae*.

**Figure 6:**
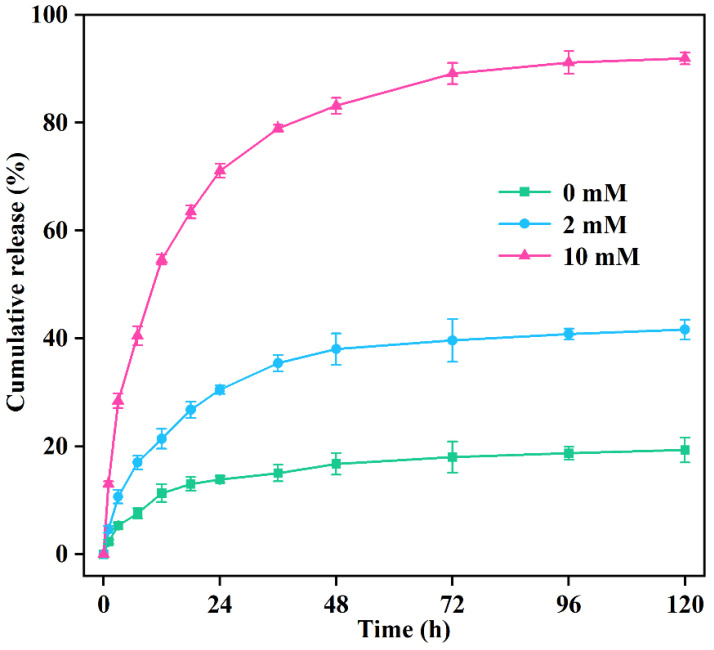
Effect of glutathione on the release performance of prochloraz from PRO@DMON–GA–Fe(III) nanoparticles.

Figure 6 shows the release manner of prochloraz from PRO@DMON–GA–Fe(III) nanoparticles in different glutathione concentrations (0, 2, and 10 mM) at room temperature. Only a small amount of prochloraz (19.34%) leaked out from PRO@DMON–GA–Fe(III) nanoparticles in the absence of glutathione after 120 h, indicating the good sealing effect of GA–Fe((III) complexes on prochloraz-loaded DMON nanocarriers. In contrast, the cumulative release amount of prochloraz increased to 41.66% at a glutathione concentration of 2 mM after 120 h. With the glutathione concentration increasing to 10 mM, the cumulative release amount of prochloraz from PRO@DMON–GA–Fe(III) nanoparticles was up to 91.96% at the same time. This redox-responsive release property is primarily ascribed to the decompositions of the GA–Fe(III) complexes and the organosilica frameworks in PRO@DMON–GA–Fe(III) nanoparticles. The results of pesticide controlled release experiment indicated that PRO@DMON–GA–Fe(III) nanoparticles could intelligently release prochloraz in response to a reductive environment produced by *Magnaporthe oryzae*.

The pesticide release is commonly controlled by diffusion or dissolution mechanisms. The experimental data were fitted using the four different kinetic models in order to further investigate the prochloraz release kinetics of PRO@DMON–GA–Fe(III) nanoparticles. As shown in Table 2, the controlled release kinetics of prochloraz were best described by the Higuchi model (*r*^2^ > 0.95). Thus, the prochloraz release was dominated by Fickian diffusion.

### 3.4. Photostability of the PRO@DMON–GA–Fe(III) Nanoparticles

**Figure 7:**
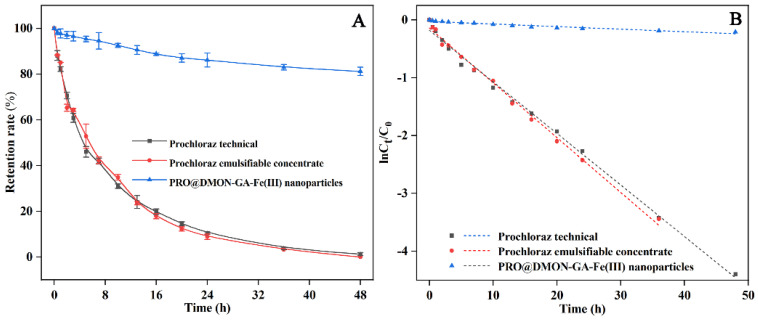
Stabilities of prochloraz technical, prochloraz emulsifiable concentrate, and PRO@DMON–GA–Fe(III) nanoparticles under UV radiation (**A**). Pseudo-first-order models of prochloraz photodegradation for prochloraz technical, prochloraz emulsifiable concentrate, and PRO@DMON–GA–Fe(III) nanoparticles (**B**).

The photostability of PRO@DMON–GA–Fe(III) nanoparticles, prochloraz emulsifiable concentrate, and prochloraz technical were evaluated under UV light. As shown in Figure 7A, prochloraz emulsifiable concentrate and prochloraz technical dispersing in deionized water decomposed rapidly after being exposed to UV irradiation. The residual rates of prochloraz in the emulsifiable concentrate and technical were only 10.36% and 8.23% after 24 h irradiation, respectively. The above results indicated that emulsifiable concentrate in the water cannot protect the active ingredients of pesticides from photolysis. In contrast, the prochloraz encapsulated with DMON–GA–Fe(III) nanoparticles exhibited better light stability, and the remaining rate of prochloraz in the nanocarriers was more than 80% after being exposed for 48 h. The photodegradation data of the PRO@DMON–GA–Fe(III) nanoparticles, prochloraz emulsifiable concentrate, and prochloraz technical could be satisfactorily fitted with the pseudo-first-order kinetics (Figure 7B). The apparent rate constants and the half-lives are fully provided in Table 3. The dissipation half-life (*DT*_50_) values of prochloraz technical, prochloraz emulsifiable concentrate, and PRO@DMON–GA–Fe(III) nanoparticles were calculated to be 7.79 h, 7.37 h, and 154.03 h, respectively. The improved photostability of the nanoparticles was attributed to the absorption or reflection of UV light by the DMON–GA–Fe(III) nanocarriers, which could reduce the absorption intensity of UV light by the loaded pesticide molecules [24].

### 3.5. Spreadability of DMON-GA-Fe(III) Nanoparticles on Rice Blades

**Figure 8:**
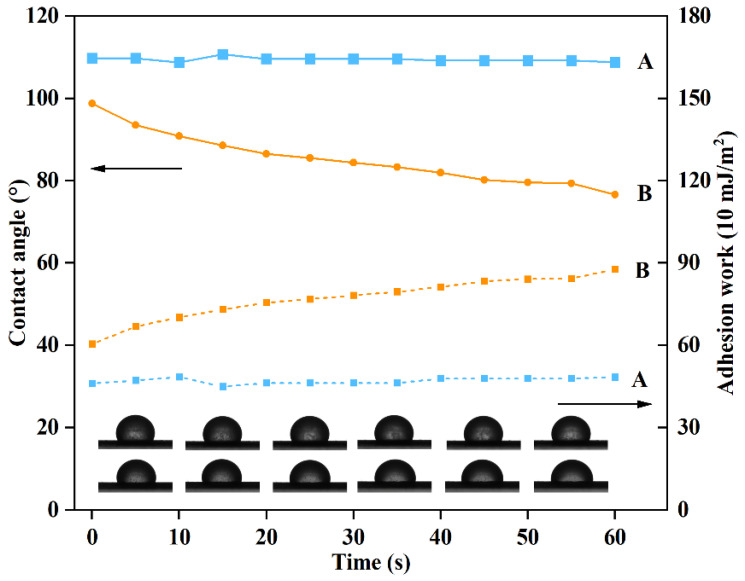
Contact angles and adhesion works of PRO@DMON nanoparticles and PRO@DMON–GA–Fe(III) nanoparticles on rice blades.

The spreadability of pesticides on the surface of plant leaves is closely related to the contact angle and adhesion work, which can effectively affect the utilization rate of active ingredients in the field [59]. The adhesion work of PRO@DMON–GA–Fe(III) nanoparticles was within 46–49 mJ/ m^2^ and the contact angle of PRO@DMON nanoparticles was within 108–110° over 60 s (Figure 8). Due to the coverage by GA–Fe(III) complex shells, PRO@DMON–GA–Fe(III) nanoparticles showed an increase in the adhesion work from 60.4 to 87.7 mJ/m^2^, while the contact angle of PRO@DMON–GA–Fe(III) nanoparticles decreased from 98.7° to 76.6° within 60 s under the same conditions. The increased adhesion work and decreased contact angle showed that PRO@DMON–GA–Fe(III) nanoparticles had a higher wettability and adhesion ability than PRO@DMON nanoparticles on the rice blades, indicating that GA–Fe(III) complex shells could effectively improve the utilization and deposition of active ingredients. The differences in the spreadability between PRO@DMON nanoparticles and PRO@DMON–GA–Fe(III) nanoparticles could be attributed to the hydrogen bonding, which is mainly generated by the interaction between the polyphenol groups of GA and fatty alcohol or fatty acid of rice leaves. The above findings were consistent with previous studies of polyphenol adhesive chemistry [60,61].

### 3.6. Bioactivity

**Figure 9:**
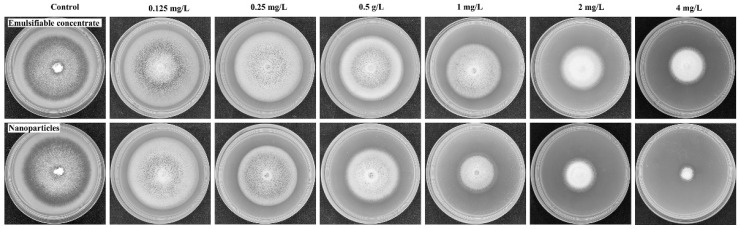
Fungicidal activities of prochloraz emulsifiable concentrate and PRO@DMON–GA–Fe(III) nanoparticles evaluated with *Magnaporthe oryzae*.

The mycelium growth rate method was adopted to investigate the fungicidal activities of prochloraz emulsifiable concentrate and PRO@DMON–GA–Fe(III) nanoparticles against *Magnaporthe oryzae*. The prochloraz emulsifiable concentrate and PRO@DMON–GA–Fe(III) nanoparticles inhibited the *Magnaporthe oryzae* colony growth in a typical dose-dependent manner (Figure 9). The coefficient of determination (*r*^2^), 95% confidence interval, *EC*_50_, and toxicity regression equations of different prochloraz formulations are shown in Table 4. Prochloraz emulsifiable concentrate and PRO@DMON–GA–Fe(III) nanoparticles had an *EC*_50_ of 2.39 and 0.81 mg/L, respectively. This indicates that PRO@DMON–GA–Fe(III) nanoparticles showed higher fungicidal activity than prochloraz emulsifiable concentrate. In addition, blank DMON–GA–Fe(III) nanoparticles did not show inhibitory effects at a concentration of 100 mg/L based on the mycelial growth diameter. Attributed to their sustained-release property and small particle size, PRO@DMON–GA–Fe(III) nanoparticles showed stronger fungicidal activity against *Magnaporthe oryzae* compared with prochloraz emulsifiable concentrate. Therefore, the prepared biodegradable nanoparticles will facilitate a highly potential approach for plant disease prevention and control in modern agriculture.

### 3.7. Safety of DMON–GA–Fe(III) Nanoparticles on Rice Seedlings 

Several physiological and biochemical parameters were used to evaluate the biosafety of DMON–GA–Fe(III) nanoparticles against rice growth [62]. These parameters sensitive to adverse environments included germination rate, fresh weight, shoot length, and root length [63]. Compared with the water (control) treatments, DMON–GA–Fe(III) nanoparticle treatments at different concentrations did not significantly affect seed germination rate, root length, shoot length, fresh weight, and root number of 7-day-old seedlings (Table 5). Therefore, these nanoparticles were non-phytotoxic, which will further improve their application to rice plant disease control in modern agriculture.

## 4. Conclusions

In this work, a redox-responsive pesticide delivery system based on metal–polyphenol-network-coated biodegradable DMON nanoparticles was established by non-covalent bonding to manage rice diseases. The prepared PRO@DMON–GA–Fe(III) nanoparticles could effectively reduce the photolysis of prochloraz by absorbing or reflecting UV light and preventing its premature leakage. In addition, the DMON–GA–Fe(III) nanocarriers with self-destruction properties could be decomposed by glutathione, realizing the on-demand release of prochloraz. The release mechanism explored by the Higuchi model presented that a Fickian diffusion controlled the release of prochloraz from PRO@DMON–GA–Fe(III) nanoparticles. The measurement of leaf contact angle and adhesion work demonstrated that GA–Fe(III) complex coating could enhance the deposition and adhesion of the nanoparticles on the rice leaves. The biological activity experiments showed that PRO@DMON–GA–Fe(III) nanoparticles had good antifungal activity and long-term efficacy against *Magnaporthe oryzae*. More importantly, the blank DMON–GA–Fe(III) nanoparticles did not show obvious toxicity to rice seedlings. Therefore, the redox-responsive biodegradable pesticide delivery system provided a promising strategy for enhancing pesticide targeting and realizing sustainable plant diseases management.

## Figures and Tables

**Table 1:** Surface and porosity properties of the samples.

Sample	BET Surface Area (m^2^/g)	Pore Volume (cm^3^/g)	Average Pore Size (nm)
DMON	1020.81	1.13	2.33
PRO@DMON	124.57	0.47	1.57
PRO@DMON–GA–Fe(III)	32.15	-	-

**Table 2:** Determined parameters of prochloraz released from PRO@DMON–GA–Fe(III) nanoparticles by fitting several kinetic equations.

Glutathione Concentration (mM)	Kinetic Model	K (×10^−2^)	*n*	*r* ^2^
0	Zero-order	0.22	—	0.7929
	First-order	0.07	—	0.7385
	Higuchi	2.17	—	0.9632
	Ritger–Peppas	3.15	0.42	0.9336
2	Zero-order	0.49	—	0.7962
	First-order	0.21	—	0.7563
	Higuchi	4.75	—	0.9646
	Ritger–Peppas	6.26	0.45	0.9385
10	Zero-order	1.10	—	0.7744
	First-order	1.44	—	0.9280
	Higuchi	10.72	—	0.9550
	Ritger–Peppas	17.46	0.39	0.9325

**Table 3:** Modeling parameters for photodegradation of prochloraz technical, prochloraz emulsifiable concentrate, and PRO@DMON–GA–Fe(III) nanoparticles under UV radiation.

Parameter	Pseudo-First-Order Kinetics		
	Technical	Emulsifiable Concentrate	Nanoparticles
*k* (h^−1^)	0.089	0.095	0.0044
*Relative index* (*r*^2^)	0.995	0.994	0.933
*DT*_50_ (h) ^a^	7.79	7.37	154.03

**Table 4:** Regression equation models obtained from prochloraz emulsifiable concentrate and PRO@DMON–GA–Fe(III) nanoparticles against *Magnaporthe oryzae*.

Treatment	Regression Equation	*EC*_50_ (95% Confidence Interval) (mg/L)	Correlation Coefficient (*r*^2^)
Prochloraz emulsifiable concentrate	y = 0.39 + 1.02x	2.39 (2.00–2.94)	0.991
PRO@DMON–GA–Fe(III) nanoparticles	y = 0.12 + 1.31x	0.81 (0.73–0.91)	0.992

*EC*_50_: Inhibitory concentration that inhibit 50% of the exposed fungus.

**Table 5:** The germination rate, root length, shoot length, root number, and fresh weight, of 7-day-old seedlings of rice germinated from seeds treated with different concentration of blank DMON–GA–Fe(III) nanoparticles.

Concentration (mg/L)	Germination Rate (%)	Root Length (cm) ^a^	Shoot Length (cm)	Root Number	Fresh Weight (mg)
0	100	7.94 ± 0.91 a	5.24 ± 0.36 a	3.54 ± 0.80 a	39.38 ± 2.37 a
50	100	8.21 ± 0.64 a	5.03 ± 0.39 a	4.04 ± 0.76 a	35.00 ± 1.02 a
100	100	7.46 ± 1.13 a	4.78 ± 0.42 a	3.45 ± 0.91 a	35.00 ± 3.68 a
200	100	7.56 ± 1.25 a	5.16 ± 0.50 a	3.67 ± 1.03 a	38.13 ± 2.58 a
400	100	8.13 ± 0.55 a	4.17 ± 0.94 a	3.83 ± 0.87 a	39.38 ± 1.57 a
800	100	7.53 ± 0.67 a	5.09 ± 0.50 a	3.27 ± 0.75 a	33.75 ± 0.72 a

^a^ Value was presented as mean ± standard deviation (n = 5). Values followed by the same letter in columns are not different at *p* < 0.05 by Duncan’s multiple range test.

## Data Availability

Not applicable.
